# Elevated DAS28, CDAI, RAPID3 and five of seven RA core data set measures in patients with positive screens for anxiety, depression or fibromyalgia on an MDHAQ

**DOI:** 10.1093/rheumatology/keaf179

**Published:** 2025-03-28

**Authors:** Theodore Pincus, Tengfei Li, Kathryn A Gibson

**Affiliations:** Division of Rheumatology, Department of Internal Medicine, Rush University School of Medicine, Chicago, IL, USA; Division of Rheumatology, Department of Internal Medicine, Rush University School of Medicine, Chicago, IL, USA; Department of Rheumatology, Liverpool Hospital, Sydney, NSW, Australia; South Western Sydney Rheumatology Research Group, Ingham Institute for Applied Medical Research, Sydney, NSW, Australia; Medicine and Health, University of New South Wales, Sydney, NSW, Australia

**Keywords:** rheumatoid arthritis, inflammation, DAS28, swollen joints, RheuMetric, anxiety, depression, fibromyalgia

## Abstract

**Objective:**

To analyze three MDHAQ (Multidimensional Health Assessment Questionnaire) patient distress screening indices, MAS2 (MDHAQ anxiety screen), MDS2 (MDHAQ depression screen) and FAST3F (fibromyalgia assessment screening tool), for potential associations with elevated rheumatoid arthritis (RA) activity/severity indices in routine care patients.

**Methods:**

A cross-sectional database included the seven RA core data set measures and three MDHAQ patient distress indices. Mean individual measures and five indices—DAS28-ESR (disease activity score 28-erythrocyte sedimentation rate), DAS28–CRP (DAS28–C-reactive protein), SDAI (simplified disease activity index), CDAI (clinical disease activity index), and RAPID3 (routine assessment of patient index data),—and the number of patients classified into high, moderate, low or remission, were computed according to positive or negative MDHAQ screening indices, including in patients with 0,1 *vs* ≥2 swollen joints, analyzed using *t* tests and chi^2^ tests.

**Results:**

Among 173 patients, positive screening was seen in 37% for MAS2, 27% for MDS2, 31% for FAST3F and 45% for at least one of these three MDHAQ screening indices. All five RA indices and five of seven core data set measures were elevated significantly (*P*-value < 0.01), other than swollen joint count (SJC), ESR and CRP, in MAS2, MDS2 and/or FAST3F positive patients, generally to a higher activity/severity category. In the 27–41% of all patients with 0 or 1 SJC but moderate/high RA index activity/severity, a positive MDHAQ anxiety, depression and/or fibromyalgia screen was seen in 54–100%.

**Conclusion:**

Five RA indices and five of seven individual core data set measures are elevated significantly in patients who screen positive for anxiety, depression and/or fibromyalgia on MDHAQ indices.

Rheumatology key messages

- Anxiety, depression, and fibromyalgia, which often are under-recognized in clinical care, may be feasibly screened for on 3 validated screening indices within a multidimensional health assessment questionnaire (MDHAQ), completed by most patients in 5–10 minutes, which agree >80% with reference standards.
- A group of 173 patients with rheumatoid arthritis (RA) who had median and mean swollen joint count of 0 and 1.7, and median and mean DAS28-CRP of 2.5 and 2,6 (indicating remission), included 37%, 27% and 31% who screened positive on the MDHAQ for anxiety, depression, and fibromyalgia, respectively, and 45% who screened positive for one of the three patient distress comorbidities.
- Positive screening for anxiety, depression, and fibromyalgia was associated with significant elevations of 5 of 7 RA core dataset measures, tender joint count, physician global assessment, physical function, pain, and patient global assessment, and elevated DAS28–ESR, DAS28–CRP, CDAI, SDAI, and RAPID3, often raising median scores from low to moderate disease activity or severity.

## Introduction

Rheumatoid arthritis (RA) is assessed for inflammatory activity by a core data set of seven measures [1], and indices derived from these measures, including Disease Activity Score 28 (DAS28) [2–4], the Simplified Disease Activity Index (SDAI) [5, 6] and Clinical Disease Activity Index (CDAI) [5, 6]. These indices and routine assessment of patient index data (RAPID3) [7, 8] on a Multidimensional Health Assessment Questionnaire (MDHAQ) [7, 9, 10], designated a *severity* rather than *activity* index [9–14], distinguish active from control treatment groups significantly in clinical trials, greatly advancing RA therapies [15]. However, RA clinical trials generally include only 5–30% of all routine care RA patients [16, 17], in part due to exclusions for comorbidities.

Routine care RA patients have a higher prevalence than the general population of anxiety (ANX) [18, 19], depression (DEP) [18–22] and fibromyalgia (FM) [22–26]. These patient distress comorbidities generally are associated with significant elevations of all six clinical RA core data set measures—least notably for swollen joint count (SJC) [22, 26], as well as of RA indices, often in the absence of inflammatory activity [20, 22, 25]. ANX, DEP and FM frequently are underrecognized without formal screening [18, 20, 21, 24], and even when recognized, often are documented only as narrative descriptions, without formal data to monitor possible changes over time. Elevated core data set measures and indices by non-inflammatory comorbidities may complicate treat-to-target strategies [27, 28], remission criteria [29, 30] and overall RA management.

Most studies concerning anxiety, depression and FM in RA have been performed in research settings rather than routine care, to recognize one or two of these comorbidities. Formal quantitative screening using multiple patient questionnaires is not feasible in busy routine care settings. As a result, structured data generally are not available to inform physician management decision at the point of care and to recognize changes over time.

**Figure 1.**
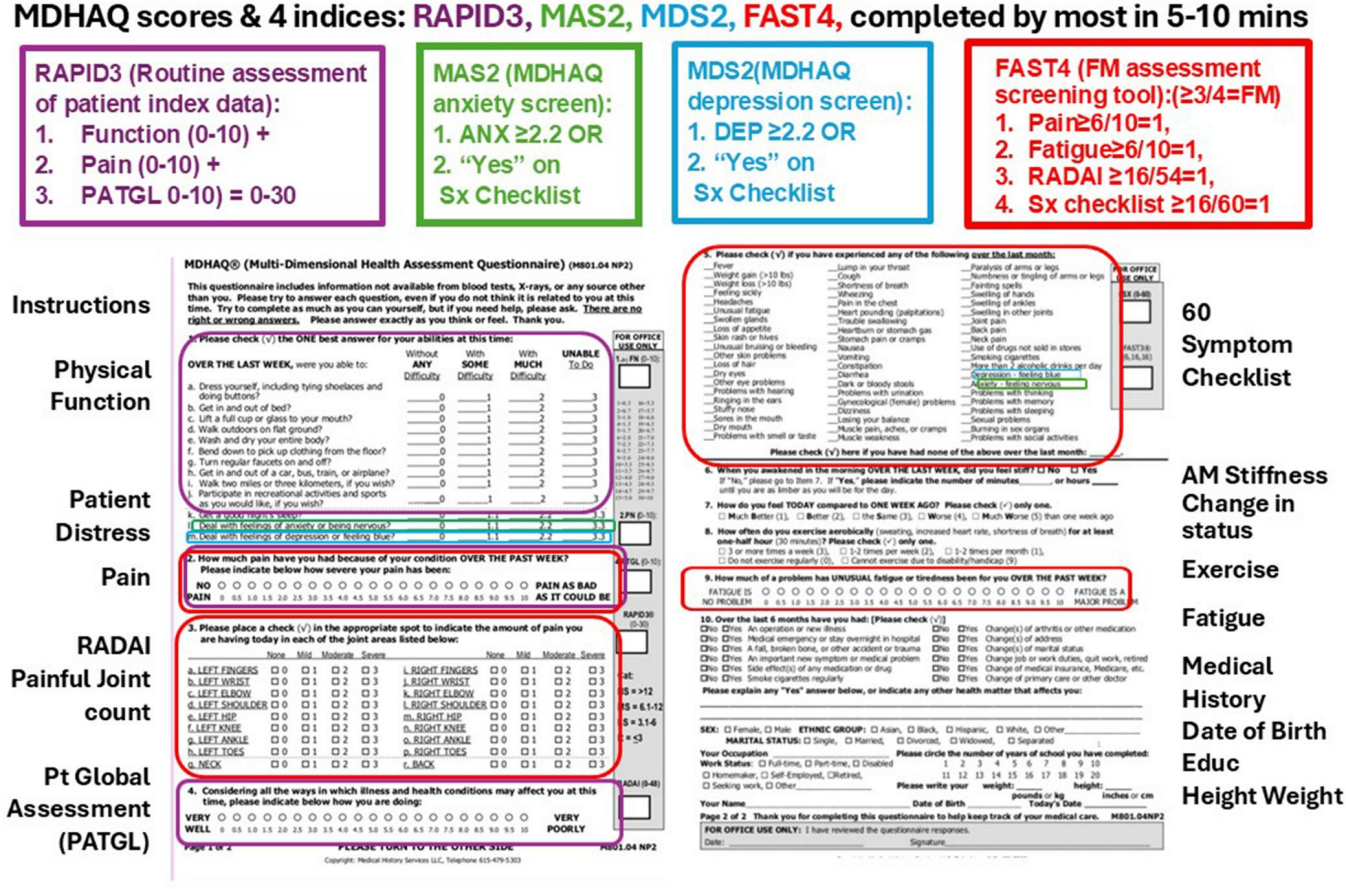
Multidimensional Health Assessment Questionnaire (MDHAQ) for routine care of any patient at any encounter

A single MDHAQ [10–14] (Fig. 1) includes three quantitative validated screening indices, MAS2 (MDHAQ anxiety screen) [14], MDS2 (MDHAQ depression screen) [13] and FAST3F (fibromyalgia assessment screening tool) [11, 12]. These indices show 80–90% agreement with the hospital anxiety and depression Scale (HADS) [13, 14, 31], patient health questionnaire-9 (PHQ-9) [13, 31, 32] and the 2011 revised FM criteria [11, 12, 33], respectively. This report presents analyses of elevations of five RA indices in routine care patients who had positive screening for anxiety, depression and/or FM on any of the three MDHAQ patient distress indices.

## Patients and methods

### Study design

An MDHAQ (Fig. 1) [7, 9, 10, 34] has been presented to all patients with all diagnosis at all routine care visit to Liverpool Hospital Rheumatology Clinic in Sydney, New South Wales, Australia since 2016 and completed by more than 80% of patients. Each rheumatologist is asked to complete a RheuMetric checklist, which includes four 0–10 visual numeric scales (VNS) estimates: physician global assessment (DOCGL), inflammation, joint damage and patient distress [35], and a 28-joint count [36] for RA patients. MDHAQ and RheuMetric data are entered into a REDCap database, hosted and managed by Research Technology Services (UNSW Sydney) as described previously [35].

Between February and June 2021, a research protocol was implemented within routine care to analyze the validity of a physician RheuMetric checklist with the four 0–10 VNS estimates in RA patients [35]. During these analyses [35], elevations of RA core data set measures and indices were observed in patients with positive screening for FM, ANX and DEP, which are analyzed in depth in this report.

### Patients

All patients were aged 18 years or older and met the 2010 American College of Rheumatology (ACR)/European Alliance of Associations for Rheumatology (EULAR) classification criteria for RA [37]. All patients gave written informed consent for use of their data detailed on the Liverpool Hospital Participant Information Sheet. Ethics approval for this study was granted by the Research and Ethics Office, South Western Sydney Local Health District (Ethics Number—SWSLHD HREC REF: LNR/13/LPOOL/370). Further information concerning these patients are available in the previous report [35].

### Measures

The patient MDHAQ (Fig. 1) [9, 10, 34], physician RheuMetric checklist [38] including a standard 28-joint count for swelling, tenderness/pain on motion and limited motion/deformity [36], ESR and CRP within 2 weeks of the consultation were entered into the RedCap database. Five RA indices, DAS28–ESR, DAS28–CRP, CDAI, SDAI and RAPID3, were computed according to standard methods [3–8].

### Multidimensional Health Assessment Questionnaire

The MDHAQ (Fig. 1) is a two‐page single‐sheet questionnaire developed from the Stanford health assessment questionnaire (HAQ) [39], based on continuous quality improvement derived from patient feedback and clinical valueutility over more than 25 years [9, 10, 34]. The MDHAQ queries 10 patient self-report scores for physical function (FN), scored as 0 (without any difficulty), 1 (with some difficulty), 2 (with much difficulty) or 3 (unable to do), as in the traditional HAQ [39], for a total of 0‐30 that is divided by 3 for a 0‐10 score [9, 10, 34]. Additional items assess sleep quality, anxiety and depression using the patient-friendly 0–3 HAQ format [9, 10, 34]. Pain, patient global assessment (PATGL) and fatigue are assessed on 0–10 VNS in increments of 0.5 unit [34]. A self-report painful joint count, modified from the 16-joint Rheumatoid Arthritis Disease Activity Index (RADAI) [40], adds neck and back for 18 joints scored 0–3, resulting in a total of 0–54 [34]. The MDHAQ also includes a 60‐symptom checklist with *Yes/No* responses to common symptoms, useful for review of systems and to identify potential adverse events to medications [41]. The MDHAQ also queries other medical history over the preceeding six months, including falls, illnesses, hospitalizations, surgeries, changes and adverse events of medications and demographic data [10, 34].

Four reported indices may be calculated from individual MDHAQ measures (Fig. 1) [34]. RAPID3 is a 0–30 composite index that includes the three patient-reported RA core data set measures: physical function (FN), pain and PATGL, each scored 0–10 [7, 8]. RAPID3 is described as a severity index rather than an activity index [7, 8], and has been found informative in assessing patient status across a range of rheumatic diseases [34, 42].

MAS2 is a cumulative index for anxiety [14], interpreted as positive if either a response ≥2 on the 0–3 anxiety query or a check for “anxiety” on the 60-symptom checklist is seen. MAS2 agrees 80.9% with the reference standard HADS-A (Hospital Anxiety and Depression Scale–Anxiety) [31]. MDS2 is a cumulative index for depression, reported initially as MDHAQ-Dep [13], based on the two MDHAQ items for depression similar to those seen in screening for anxiety on MAS2 (Fig. 1). MDS2 agrees 81.7–83.3% with the reference PHQ-9 (patient health questionnaire 9) [32] and HADS-D (hospital anxiety and depression scale–Depression) [31], closely matching the 82.2% agreement of the two reference questionnaires with one another [13].

FAST4 [11, 12] yields a positive FM screen, which agrees approximately 90% with the 2011 revised FM criteria [33], if three of four variables are positive: pain VNS ≥ 6/10, fatigue VNS ≥ 6/10, self-report joint count ≥ 16/54 and 60 symptom checklist ≥ 16/60. FAST3F, excludes pain from FAST4, gives approximately 90% agreement with 2011 revised FM criteria [33], and appeared appropriate for this study, as patient pain VNS is correlated highly significantly with PATGL VNS, which is included in all the RA activity/severity indices (r = 0.85 in the database analyzed in this report) [35].

### Statistical analyses

Data from the REDCap database were transferred as a de-identified Excel spreadsheet for analyses at Rush University using R and SAS. Among the 173 patients, 142 had complete data on MDS2, MAS2 and FAST3F; missing data in 31 patients were generally ESR, CRP and/or select MDHAQ items. Analyses of all 173 *vs* 142 with missing data showed no meaningful differences. Therefore, demographic characteristics and joint counts are reported for all 173 patients, while all other analyses are applied to the 142 patients with minimal missing data.

Descriptive statistics were computed as mean and standard deviation (SD) and median and interquartile range (IQR) of continuous variables, and as counts and percentages of categorical variables, including demographic characteristics, RA core dataset measures and RA indices, DAS28–ESR, DAS28-CRP, CDAI, SDAI and RAPID3, as well as MDHAQ-based patient distress screens, MAS2, MDS2 and FAST3F (as well as FAST4). Mean RA index scores were compared in patients whose screening was negative or positive for MAS2, MDS2 and FAST3F, and between those with all three negative screens or any positive screen, analyzed for statistical significance using t tests. Cross- tabulations were computed for each RA index classified in two categories, remission(R)/low(L) and moderate(M)/high (H), *vs* negative or positive MAS2, MDS2, FAST3F, all three negative *vs* any positive screen, analyzed using chi^2^ tests. Statistical significance was established if corresponding two-sided *P*-value was <0.05. Further analyses were conducted identically in patients with 0 or 1 SJC vs those with SJC ≥ 2, for two categories of RA disease activity/severity indices *vs* a positive or negative MAS2, MDS2, FAST3F, all three negative *vs* any positive MDHAQ patient distress screen.

## Results

### Demographic, joint count, RA indices, and MDHAQ screening indices

The study included 173 unselected routine care RA patients. Mean and median ages were 59.5 and 61.0 years, respectively; mean and median disease duration were 11.7 and 10.0 years, respectively; 75% of patients were female (Table 1). Mean and median SJC were 1.7 and 0, TJC 3.8 and 2.0, and DJC 5.4 and 4.0, respectively; 71% of patients had SJC 0,1, 47% TJC 0,1, and 38% DJC 0,1, respectively (Table 1). Mean and median DAS28–ESR were 3.0 (low activity=L) and 2.9 (L), DAS28–CRP 2.6 (remission=R) and 2.5 (R), CDAI 13.8 (moderate=M) and 12.0 (M), SDAI 19.2 (M) and 17.0 (M), and RAPID3 11.6 (M) and 12.0 (M), respectively (Table 1). Positive screening for anxiety on MAS2 was seen in 37% of the patients 27% for depression on MDS2, 31% for FM on FAST3F, and 45% for anxiety, depression and/or FM (Table 1)—Two MDHAQ indices were positive in 15% of patients and all three in 18%.

**Table 1.** Measures and indices in 173 study patients with rheumatoid arthritis at Liverpool Hospital

Measure [Units, range or Number (%)]	Mean (SD)	Median (IQR)
Total number of patients	173	—
Age (years)	59.5 (14.3)	61.0 (50.5-70.0)
Disease duration (years)	11.7 (9.3)	10.0 (6-15)
Number (%) female	129 (75%)	—
Swollen joint count (SJC) (0-26)	1.7 (2.9)	0 (0-2.0)
Number (%) with SJC 0,1	123 (71%)	—
Tenderness/pain on motion joint count (TJC) (0-28)	3.8 (5.7)	2.0 (0-5.0)
Number (%) with TJC 0,1	82 (47%)	—
Deformed/limited motion joint count (DJC) (0-28)	5.4 (6.3)	4.0 (0-9.0)
Number (%) with DJC 0,1	65 (38%)	—
Physician global assessment (DOCGL) (0-10)	3.7 (2.4)	3.5 (1.5-6)
Patient global assessment (PATGL) (0-10)	4.5 (3.0)	4.5 (1.5-7)
Erythrocyte sedimentation rate (ESR) (mm/h)	22.0 (19.2)	17.5 (8.0-29.0)
Number (%) ESR in males > 20 mm/h or females > 30 mm/h	43/159 (27%)	—
C-reactive protein (CRP) (mg/L)	5.4 (6.8)	3.8 (1.1-7)
Number (%) CRP ≥10 mg/L	22/162 (14%)	—
Number (%) positive FM FAST3F screen (MAS2 and MDS2 pos/neg)	44/142 (31%)	
Number (%) positive ANX MAS2 screen (MDS2 and FAST3F pos/neg	53/142 (37%)	
Number (%) positive DEP MDS2 screen (MAS2 and FAST3F pos/neg	39/142 (27%)	
Number (%) positive FAST3F or MAS2 or MDS2 screen	64/142 (45%)	
DAS28-ESR (Disease activity score 28) (18 missing)	3.0 (1.2)	2.9 (2.2-3.7)
Number (% of non-missing) DAS28-ESR remission	67/155 (43%)	
Number (% of non-missing) DAS28-ESR low activity	27/155 (17%)	
Number (% of non-missing) DAS28-ESR moderate activity	51/155 (33%)	
Number (% of non-missing) DAS28-ESR high activity	10/155 (6%)	
DAS28-CRP (Disease activity score 28) (16 missing)	2.6 (1.0)	2.5 (1.9-3.1)
Number (% of non-missing) DAS28-CRP remission	90/157 (57%)	
Number (% of non-missing) DAS28-CRP low activity	34/157 (22%)	
Number (% of non-missing) DAS28-CRP moderate activity	31/157 (20%)	
Number (% of non-missing) DAS28-CRP high activity	2/157 (1%)	
CDAI (clinical disease activity index) (5 missing)	13.8 (10.6)	12.0 (5.5-18.5)
Number (% of non-missing) CDAI remission	20/168 (12%)	
Number (% of non-missing) CDAI low activity	50/168 (30%)	
Number (% of non-missing) CDAI moderate activity	71/168 (42%)	
Number (% of non-missing) CDAI high activity	27/168 (16%)	
SDAI (simplified disease activity index) (16 missing)	19.2 (12.6)	17.0 (10.0-24.4)
Number (% of non-missing) SDAI remission	8/157 (5%)	
Number (% of non-missing) SDAI low activity	34/157 (22%)	
Number (% of non-missing) SDAI moderate activity	81/157 (52%)	
Number (% of non-missing) SDAI high activity	34/157 (22%)	
RAPID3 (Routine assessment of patient index data) (0-30) (12 missing)	11.6 (7.2)	12.0 (4.7-17.2)
Number (% of non-missing) RAPID3 remission	27/161 (17%)	
Number (% of non-missing) RAPID3 low severity	25/161 (16%)	
Number (% of non-missing) RAPID3 moderate severity	27/161 (17%)	
Number (% of non-missing) RAPID3 high Severity	82/161 (51%)	

### Mean RA indices and individual RA core data set measures in patients who screened positive or negative for MAS2 (anxiety), MDS2 (depression), FAST3F (FM) or any of the three MDHAQ screens

All five RA indices studied, DAS28-ESR, DAS28-CRP, CDAI, SDAI and RAPID3, differed significantly according to a negative *vs* positive screen for MAS2, MDS2, FAST3F or any of the three indices (Table 2). If all three screens were negative *vs* any positive, DAS28-ESR was 2.7 (L) *vs* 3.3 (M), DAS28 CRP 2.3 (R) *vs* 3.1 (L), CDAI 8.6 (L) *vs* 20.7 (M), SDAI 14.5 (M) *vs* 25.5 (M), and RAPID3 7.2 (M) *vs* 16.9 (H) (Table 2). All differences were clinically relevant and statistically significant (*P*-value < 0.01); a positive screen was generally associated with mean elevations of the index by one category (Table 2).

**Table 2.** Mean (standard deviation) of five RA indices and eight individual core RA core data set measures according to a negative screen on all 3 MDHAQ patient distress indices, FAST3F, MAS2 and MDS2 *vs* one or more with a positive screen

Measure (range/units)	Mean of all patients	FAST3F–	FAST3F+	MAS2−	MAS2+	MDS2−	MDS2+	FAST3F− & MDS2− & MAS2−	FAST3F+ or MDS2+ or MAS2+

Number of patients	142	98 (69%)	44 (31%)	89 (63%)	53 (37%)	103 (73%)	39 (27%)	78 (55%)	64 (45%)

**a. 5 RA indices**									
**DAS28-ESR**	3.0 (1.2)L	2.7 (1.1) L	3.5 (1.3)M***	2.7 (1.1) L	3.5 (1.3) M ***	2.7 (1.1) L	3.6 (1.3) M**	2.7 (1.1) L	3.3 (1.3) M **
**DAS28CRP**	2.6 (1.0)R	2.4 (0.9) R	3.2 (1.1) L****	2.3 (0.8) R	3.2 (1.1) L****	2.4 (0.9) R	3.2 (1.1) L***	2.3 (0.8) R	3.1 (1.1) L****
**CDAI**	14.1 (11.0)M	9.9 (7.9) L	23.3 (11.1)H ****	9.7 (7.2) L	21.4 (12.2)M ****	11.0 (8.6) M	22.0 (12.5)M ****	8.6 (6.6) L	20.7 (11.6)M ****
**SDAI**	19.4 (12.9)M	15.5 (10.4) M	28.4 (13.7) H****	15.3 (9.9)M	26.5 (14.7)M ****	16.4 (10.5)M	27.7 (15.5)H***	14.5 (9.9) M	25.5 (13.7) M****
**RAPID3**	11.6 (7.3)M	8.0 (5.3) M	19.5 (4.5)H****	8.3 (5.8) M	17.2 (6.3) H****	9.4 (6.6) M	17.5 (5.8) H****	7.2 (5.1) M	16.9 (5.9) M****

**b. Individual RA core data set **measures									
SJC 26	1.7 (3.0)	1.6 (2.8)	1.9 (3.4) ^^^	1.2 (2.2)	2.5 (3.9) *	1.3 (2.3)	2.8 (4.2) *	1.3 (2.3)	2.3 (3.6)^^^
TJC 28	4.0 (6.0)	2.3 (3.7)	7.8 (8.1)****	2.1 (3.4)	7.2 (7.8)****	2.8 (4.6)	7.0 (8.0)**	1.8 (2.4)	6.7 (7.8)****
PATGL	4.5 (3.0)	3.1 (2.5)	7.6 (1.6)****	3.3 (2.7)	6.5 (2.5)****	3.7 (2.9)	6.6 (2.2)****	2.8 (2.5)	6.6 (2.3)****
ESR	21.0 (18.8)	22.1 (20.6)	18.5 (13.7)^^^	22.8 (21.8)	17.7 (11.5)^^^	21.8 (20.6)	18.6 (12.2)^^^	23.3 (22.1)	18.0 (13.0)^^^
CRP	5.3 (6.7)	5.4 (6.8)	4.9 (6.7)^^^	5.5 (7.0)	4.9 (6.3)^^^	5.3 (6.6)	5.4 (7.1)^^^	5.7 (7.4)	4.7 (5.8)^^^
DOCGL	3.6 (2.4)	2.8 (2.0)	5.6 (2.1)****	2.9 (2.1)	4.9 (2.5)****	3.0 (2.1)	5.2 (2.4)****	2.5 (1.9)	5.0 (2.3)****
Function	2.5 (2.1)	1.6 (1.4)	4.5 (1.9)****	1.6 (1.5)	4.1 (2.1)****	1.9 (1.7)	4.2 (2.2)****	1.4 (1.3)	3.9 (2.0)****
Pain	4.6 (2.8)	3.3 (2.3)	7.4 (1.8)****	3.4 (2.4)	6.5 (2.4)****	3.8 (2.7)	6.6 (2.1)****	3.0 (2.2)	6.4 (2.4)****

Data with missing FAST3F, MAS2, MDS2 category or the measure was excluded from the analysis for the specific measure. FAST3F: fibromyalgia assessment screening tool; MAS2: MDHAQ anxiety screen; MDS2: MDHAQ depression screen.

^ *P* > 0.05.

* *P* < 0.05.

** *P* < 0.01.

*** *P* < 0.001.

**** *P* < 0.0001.

Mean values of five of the seven RA core data set measures, TJC, DOCGL, PATGL, FN, and pain VNS, differed clinically and statistically significantly (*P* < 0.0001 in most comparisons) between patients who screened negative *vs* positive for FAST3F, MAS2, MDS2 or any of the three indices (Table 2). Mean SJC was statistically significantly higher in patients who screened positive *vs* negative for MAS2 and MDS2 (*P* < 0.05), but differences according to FAST3F or any index were not significant. More importantly, differences between negative and positive MDHAQ patient distress screens ranged from 0.3 to 1.5 for SJC, compared to 4.2 to 5.5 for TJC, indicating clinically meaningful differences between the two joint count measures (Table 2). Differences in ESR or CRP in patients with negative *vs* positive screens were not significant, as anticipated.

### Proportion of patients classified as remission/low (R/L) *vs* moderate/high (M/H) activity/severity on RA indices by MDHAQ anxiety, depression or FM screens

Remission/low activity/severity was seen in 26–79% of patients according to DAS28–ESR, DAS28–CRP, CDAI, SDAI and RAPID3, respectively (Table 3, column b). Patients classified as in remission/low activity/severity included 63–93% with all negative screens for FAST3F, MAS2 and MDS2 (Table 3, column i), compared to 55–71% with any positive screen (Table 3, column j). Nonetheless, 29–45% of patients who screened negative for any index were classified as in moderate/high activity/severity, while 7–37% of patients with positive screens were in remission/low activity/severity (Table 3, columns i and j); all differences were statistically significant (*P*-value < 0.01).

**Table 3.** Number of patients for five RA indices *vs* categories of remission/low *vs* moderate/high on five RA indices, according to a negative screen on all three MDHAQ patient distress indices, FAST3F (fibromyalgia), MAS2 (anxiety) and MDS2 (depression) *vs* a positive screen on one or more index

a. Index total & remission/low (R/L) & moderate/high (M/H)	b. Total (column%)	c. FAST3F–(row %)	d. FAST3F+ (row %)	e. MAS2− (row %)	f. MAS2+(row %)	g. MDS2−(row %)	h. MDS2+(row %)	i.FAST3F− & MDS2− & MAS2−(row%)		**j. FAST3F+ or MDS2+ or** **MAS2+ (row%)**
**DAS28-ESR**	129	89 (69%)	40 (31%)	83 (64%)	46 (36%)	95 (74%)	34 26%)	72 (56%)		57 (44%)
**R/L**	78 (60%)	62 (79%)	16 (21%)	58 (74%)	20 (26%)	65 (83%)	13 (17%)	51 (65%)		27 (35%)
**M/H**	51 (40%)	27 (53%)	24 (47%)	25 (49%)	26 (51%)	30 (59%)	21 (41%)	21 (41%)		30 (59%)
**Chi^2^, *P***		Chi^2^ = 10.2, *P* < 0.005		Chi^2^ = 8.6, *P* < 0.005		Chi^2^ = 9.5, *P* < 0.005			Chi^2^ = 7.3, *P* < 0.01	
**DAS28-CRP**	131	91 (69%)	40 (31%)	84 (64%)	47 (36%)	97 (74%)	34 (26%)	73 (56%)		58 (44%)
**R/L**	103 (79%)	79 (77%)	24 (23%)	74 (72%)	29 (28%)	82 (80%)	21 (20%)	65 (63%)		38 (37%)
**M/H**	28 (21%)	12 (43%)	16 (57%)	10 (36%)	18 (64%)	15 (54%)	13 (46%)	8 (29%)		20 (71%)
**Chi^2^, *P***		Chi^2^ = 11.9, *P* < 0.001		Chi^2^ = 12.5, *P* < 0.0005		Chi^2^ = 7.8, *P* < 0.01			Chi^2^ = 10.6, *P* < 0.005	
**CDAI**	141	97 (69%)	44 (31%)	88 (62%)	53 (38%)	102 (72%)	39 (28%)	77 (55%)		64 (45%)
**R/L**	61 (43%)	59 (97%)	2 (3%)	50 (82%)	11 (18%)	55 (90%)	6 (10%)	50 (82%)		11 (18%)
**M/H**	80 (57%)	38 (48%)	42 (53%)	38 (48%)	42 (53%)	47 (59%)	33 (41%)	27 (34%)		53 (66%)
**Chi^2^, *P***		Chi^2^ = 39.1, *P* < 0.0001		Chi^2^ = 17.5, *P* < 0.0001		Chi^2^ = 17.1, *P* < 0.0001			Chi^2^ = 32.5, *P* < 0.0001	
**SDAI**	131	91 (69%)	40 (31%)	84 (64%)	47 (36%)	97 (74%)	34 (26%)	73 (56%)		58 (44%)
**R/L**	34 (26%)	33 (97%)	1 (3%)	29 (85%)	5 (15%)	31 (91%)	3 (9%)	29 (85%)		5 (15%)
**M/H**	97 (74%)	58 (60%)	39 (40%)	55 (57%)	42 (43%)	66 (68%)	31 (32%)	44 (45%)		53 (55%)
**Chi^2^, *P***		Chi^2^ = 16.5, *P* < 0.0001		Chi^2^ = 9.0, *P* < 0.005		Chi^2^ = 7.0, *P* < 0.01			Chi^2^ = 16.3, *P* < 0.0001	
**RAPID3**	137	94 (69%)	43 (31%)	86 (63%)	51 (37%)	99 (72%)	38 (28%)	75 (55%)		62 (45%)
**R/L**	46 (34%)	46 (100%)	0 (0%)	43 (93%)	3 (7%)	45 (98%)	1 (2%)	43 (93%)		3 (7%)
**M/H**	91 (66%)	48 (53%)	43 (47%)	43 (47%)	48 (53%)	54 (59%)	37 (41%)	32 (35%)		59 (65%)
**Chi^2^, *P***		Chi^2^ = 31.7, *P* < 0.0001		Chi^2^ = 27.9, *P* < 0.0001		Chi^2^ = 22.6, *P* < 0.0001			Chi^2^ = 41.9, *P* < 0.0001	

Data with missing FAST3F, MAS2, MDS2 category or the measure were excluded from the analysis for the specific measure. R/L: remission/low; M/H: moderate/high.

### Analyses of patients with 0,1 swollen 28 joint counts (SJC) in five RA index categories of remission/low (R/l) or moderate/high (M/H) vs negative or positive MDHAQ anxiety, depression and/or FM screens

Overall, 69–71% of the patients had an SJC of 0,1 (proportions differed slightly for different indices as totals varied for each index), meeting the SJC criterion for remission [29, 30] (Table 4, column c). Nonetheless, 21–74% of all patients were in M/H activity/severity according to DAS28–ESR, DAS28–CRP, CDAI, SDAI and RAPID3, respectively (Table 4, column b).

**Table 4.** Number of patients with 0 or 1 28-swollen joint count *vs* categories of remission/low *vs* moderate/high on five RA indices, according to a negative screen on all three MDHAQ patient distress indices, FAST3F (fibromyalgia), MAS2 (anxiety) and MDS2 (depression) *vs* a positive screen on one or more index

a. Index total & remission/low R/L) or moderate/high (M/H)	b. Total (column % of all patients)	c. Total SJC 0,1 (row % of column b)	d. SJC0,1 FAST3F– MDS2−MAS2– (row % of column c)	e. SJC 0,1 FAST3F+ and/or MDS2+ and/or MAS2+ (row % of column c)
**DAS28-ESR**	129	91 (71%)	55 (60%)	36 (40%)
**R/L**	78 (60%)	67 (86%)	45 (67%)	22 (33%)
**M/H**	51 (40%)	24 (47 %)	10 (42%)	14 (58%)
***P*: col d *vs* e**				0.03
**DAS28-CRP**	131	92 (70%)	55 (60%)	37 (40%)
**R/L**	103 (79%)	84 (82%)	55 (65%)	29 (35%)
**M/H**	28 (21%)	8 (29%)	0 (0%)	8 (100%)
***P*: col d *vs* e**				0.0003
**CDAI %**	141	98 (70%)	58 (59%)	40 (41%)
**R/L (%)**	61 (43%)	55 (90%)	46 (84%)	9 (16%)
**M/H (%)**	80 (57%)	43 (54%)	12 (28%)	31 (72%)
***P*: col d *vs* e**				<0.0001
**SDAI**	131	92 (70%)	55 (60%)	37 (40%)
**R/L**	34 (26%)	31 (91%)	27 (87%)	4 (13%)
**M/H**	97 (74%)	61 (63%)	28 (46%)	33 (54%)
***P*: col d *vs* e**				0.0001
**RAPID3**	137	95 (69%)	56 (59%)	39 (41%)
**R/L**	46 (34%)	35 (76%)	33 (94%)	2 (6%)
**M/H**	91 (66%)	60 (66%)	23 (38%)	37 (62%)
***P*: col d *vs* e**				<0.0001

SJC: swollen joint count; R/L: remission/low; M/H: moderate/high.

SJC 0,1 was seen in 76–91% of patients classified as R/L according to DAS28–ESR, DAS28–CRP, CDAI, SDAI or RAPID3, as expected (Table 4, column c), although 29–66% of patients classified as M/H according to each of the five RA indices also had SJC 0,1 (Table 4, column c). Among patients with SJC 0,1 and negative MDHAQ screens for anxiety, depression and/or FM, 65–94% were classified as R/L by DAS28–ESR, DAS28–CRP, CDAI, SDAI or RAPID3 (Table 4, column d). Patients with SJC 0,1 and positive MDHAQ screens for anxiety, depression and/or FM included 54–100% who were classified as M/H by DAS28–ESR, DAS28–CRP, CDAI, SDAI or RAPID3 (Table 4, column e) (Table 4, column d) (*P*-value = 0.03 to <0.0001).

### Analyses of patients with 0,1 *vs* ≥2 swollen 28 joint counts (SJC) in remission/low (R/L) *vs* moderate/high (M/H) for five RA indices according to negative *vs* positive MDHAQ anxiety, depression and/or FM screens

As expected, patients with SJC 0,1 *vs* ≥2 were more likely to be in R/L *vs* M/H for all RA five indices (Table 5, columns c and d), although differences were not statistically significant for RAPID3. Patients with SJC 0,1 and any positive MDHAQ patient distress screen included 27–41% who were classified as H/M on DAS28–ESR, DAS28–CRP, CDAI, SDAI or RAPID3 (Table 5, column f). Patients whose three MDHAQ patient distress screens were all negative were more likely to be in R/L if SJC was 0,1 rather than ≥2 across all five RA indices (Table 5, columns e and g), again not statistically significant for RAPID3. Among patients with SJC ≥2, differences between those with no positive MDHAQ patient distress screens *vs* any positive screen were not statistically significant, except for RAPID3 (Table 5, columns g and h).

**Table 5.** Number of patients with 0,1 or ≥2 swollen joints on a 28 joint count *vs* categories of remission/low *vs* moderate/high on five RA indices, according to a negative screen on all three MDHAQ patient distress indices, FAST3F (fibromyalgia), MAS2 (anxiety) and MDS2 (depression) *vs* a positive screen on one or more index

a. Index total & R/L or M/H	b. Total (column % of all patients)	c. Total SJC 0,1 (row % of column b)	d. Total SJC ≥2 (row % of column b	e. SJC0,1 FAST3F− & MDS2− & MAS2− (row % of column b)	f. SJC 0,1 & (FAST3F+ or MDS2+ or MAS2+) (row % of column b)	g. SJC ≥2 & FAST3F− & MDS2− & MAS2− (row % of column b)	h. SJC ≥2 & (FAST3F+ or MDS2+ or MAS2+) (row % of column b)
**DAS28-ESR**	129	91 (71%)	38 (29%)	55 (43%)	36 (28%)	17 (13%)	21 (16%)
**R/L**	78 (60%)	67 (86%)	11 (14%)	45 (58%)	22 (28%)	6 (8%)	5 (6%)
**M/H**	51 (40%)	24 (47 %)	27 (53%)	10 (20%)	14 (27%)	11 (22%)	16 (31%)
***P*: c *vs* d SJC 0,1 *vs* ≥2**			<0.0001				
***P*: e *vs* g SJC0,1 *vs* ≥2 all-**						0.0002	
***P*: g *vs* h SJC ≥ 2 any+ *vs* all-**							0.44
**DAS28-CRP**	131	92 (70%)	39 (30%)	55 (42%)	37 (28%)	18 (14%)	21 (16%)
**R/L**	103 (79%)	84 (82%)	19 (18%)	55 (53%)	29 (28%)	10 (10%)	9 (9%)
**M/H**	28 (21%)	8 (29%)	20 (71%)	0 (0%)	8 (29%)	8 (29%)	12 (43%)
***P*: c *vs* d SJC 0,1 *vs* ≥2**			<0.0001				
***P*: e *vs* g SJC0,1 *vs* ≥2 all-**						<0.0001	
***P*: g *vs* h SJC ≥ 2 any+ *vs* all-**							0.43
**CDAI %**	141	98 (70%)	43 (31%)	58 (41%)	40 (28%)	19 (13%)	24 (17%)
**R/L (%)**	61 (43%)	55 (90%)	6 (10%)	46 (75%)	9 (15%)	4 (7%)	2 (3%)
**M/H (%)**	80 (57%)	43 (54%)	37 (46%)	12 (15%)	31 (39%)	15 (19%)	22 (28%)
***P*: c *vs* d SJC 0,1 *vs* ≥2**			<0.0001				
***P*: e *vs* g SJC0,1 *vs* ≥2 all-**						<0.0001	
***P*: g *vs* h SJC ≥ 2 any+ *vs* all-**							0.23
**SDAI**	131	92 (70%)	39 (30%)	55 (42%)	37 (28%)	18 (14%)	21 (16%)
**R/L**	34 (26%)	31 (91%)	3 (9%)	27 (79%)	4 (12%)	2 (6%)	1 (3%)
**M/H**	97 (74%)	61 (63%)	36 (37%)	28 (29%)	33 (34%)	16 (16%)	20 (21%)
***P*: c *vs* d SJC 0,1 *vs* ≥2**			0.002				
***P*: e *vs* g SJC0,1 *vs* ≥2 all-**						0.004	
***P*: g *vs* h SJC ≥ 2 any+ *vs* all-**							0.46
**RAPID3**	137	95 (69%)	42 (31%)	56 (41%)	39 (28%)	19 (14%)	23 (17%)
**R/L**	46 (34%)	35 (76%)	11 (24%)	33 (72%)	2 (4%)	10 (22%)	1 (2%)
**M/H**	91 (66%)	60 (66%)	31 (34%)	23 (25%)	37 (41%)	9 (10%)	22 (24%)
***P*: c *vs* d SJC 0,1 *vs* ≥2**			0.22				
***P*: e *vs* g SJC0,1 *vs* ≥2 all-**						0.63	
***P*: g *vs* h SJC ≥ 2 any+ *vs* all-**							0.0004

SJC: swollen joint count; R/L: remission/low; M/H: moderate/high.

## Discussion

The findings in this report confirm prior evidence that five of seven RA core data set measures and DAS28-ESR are elevated significantly in patients who screen positive for anxiety, depression or FM [18–25]. This study extends the existing knowledge in several respects: (a) Anxiety, depression and FM were screened for on a single MDHAQ, completed by most patients in 5–10 minutes in either an electronic or paper format [43], in contrast to previous studies, which typically involved multiple questionnaires, and few (if any to our knowledge) assessed all three comorbidities in the same RA patients. (b) MDHAQ screening data were collected at routine care encounters and available to the clinicians at the point of care, rather than in research settings, where screening data were not available for active clinical decisions. (c) Similar elevations of RA measures and indices to DAS28-ESR are presented according to four additional RA indices, DAS28–CRP, CDAI, SDAI and RAPID3. (d) Among patients with SJC 0,1 who had positive MDHAQ anxiety, depression or FM screens, 54–100% were classified as in moderate/high activity/severity according to DAS28–CRP, CDAI, RAPID3, DAS28–ESR or SDAI (Table 4). These findings in routine settings might inform clinical decisions concerning treat-to-target strategies [27, 28], remission criteria [29, 30] and overall RA patient management. patients 

The MDHAQ screening indices were developed from routine care data at different sites: MAS2 for anxiety in Liverpool (Sydney) Australia [14], MDS2 for depression in Barcelona, Spain [13], and FAST4 in Chicago, Illinois, USA [11] (and later also in Liverpool [12]). Each site collected the MDHAQ with one or two additional reference questionnaires. As noted, completing three additional reference questionnaires was not feasible, even in settings at which patient questionnaires are a component of routine care. Five RA activity/severity indices were studied primarily to ascertain whether associations of any index with a positive MDHAQ anxiety, depression and/or FM screen were generalizable, rather than to compare the indices in detail. However, some differences appear relevant. The mean DAS28–CRP in all patients of 2.6 indicated remission, an unusual finding in the rheumatology literature, reflecting that DAS28-CRP generally indicates better clinical status than DAS28–ESR, although the formal activity categories remain identical for both versions of DAS28 [4]. The mean DAS28-ESR of 3.0 was classified as low activity, while the mean CDAI of 13.8 and SDAI of 19.2 were classified as moderate activity and the mean RAPID3 of 11.6 indicated moderate severity. RAPID3, which is composed of solely patient self-report data, was the most elevated index among all five RA indices by the three MDHAQ patient distress indices, as anticipated. Nonetheless, among patients with SJC 0,1 and a positive MDHAQ screen for anxiety, depression or FM, 72% were in M/H for CDAI *vs* 62% for RAPID3, i.e. were 39% and 41% of all M/H patients by CDAI and RAPID3, respectively (Table 5). The primary message of this report is that all five indices are elevated in patients who screen positive on any of the MDHAQ patient distress indices.

RA indices derived from a core data set [1] provide documented value in clinical trials, in which recognized conditions such as anxiety, depression and FM often are exclusions from patient enrollment. In clinical trials, even if unrecognized or underrecognized [12, 20, 22, 35], a similar number of patients with each or any of these patient distress comorbidities would likely be randomized across trial arms. This could result in a similar potential confounding effect on results. However, clinical trials represent only a small fraction of RA patients, generally 5–30% [16, 17], and most RA patients in routine care differ from those seen in clinical trials.

In this report, 30%, 35%, 27% and 44% of patients screened positive on MDHAQ screens for anxiety, depression, FM or any of the three patient distress comorbidities, respectively, suggesting that these diagnoses are common among routine care rheumatology patients, albeit typically as secondary comorbidities. These comorbidities may not have been as prominently recognized prior to 2000 when powerful new biological agents greatly enhanced control of inflammatory activity—leaving relatively few earlier studies available. The increasing attention to patient distress comorbidities may partly reflect the improved control of inflammatory activity at this time [15].

The need to interpret an elevated RA index in patients with anxiety, depression and/or fibromyalgia does not undermine the validity and usefulness of these indices in routine care. Interpretation of an elevated RA index by a patient distress index on the MDHAQ may be analogous to interpretation of an elevated ESR, which may indicate an infection or neoplasm rather than inflammatory activity in certain patients. Several reports indicate non-intensification of therapy in many patients with M/H activity/severity index values according to treat-to-target strategy [44, 45] recognizing that such (in)actions based on a shared decision may be appropriate for some patients [27, 28]. Documentation of a positive screen for anxiety, depression or FM in 58–100% of patients who have 0,1 SJC and M/H index status (Table 4), who constitute 27–41% of all patients with M/H indices (Table 5), may provide an improved explanation concerning non-escalation of therapy in patients with moderate/high indices than simple narrative descriptions.

This study has many limitations. First, only 173 patients at a single institution were studied, although the patients appear typical of contemporary patients with a disease duration of 10 years whose inflammatory activity are well-controlled. Second, the observation that >40% of patients screen positive for anxiety, depression or FM may not be fully generalizable, although the literature [18–25] and other reports from Barcelona, Chicago, and Liverpool (some patients in the Liverpool database were in the current database) with similar findings [46, 47] suggest moderate generalizability. Third, the study is cross-sectional, without information concerning whether anxiety, depression or FM may change, possibly associated with or independent of improvement or worsening of RA status. Fourth, patients had a low level of inflammatory activity, and findings may differ in patient groups enriched for inflammatory activity (as in a clinical trial). Fifth, the data present only screening information, as a formal diagnosis of anxiety, depression or FM requires evaluation by a trained healthprofessional, although the reference questionnaires to establish validity of the MDHAQ screens indicated correlations with formal health professional diagnoses.

In conclusion, validated screening indices for anxiety, depression or FM embedded within a single MDHAQ were positive in 44% of routine care RA patients, associated with M/H index activity/severity categories in 54–100% of patients with SJC 0,1, who were 27–41% of all M/H patients. Anxiety, depression and FM are more common in RA than in the general population, but often underrecognized or unrecognized [20, 33]. Even when recognized, these comorbidities are usually recorded only as narrative descriptions without structured data to track possible change. We suggest screening all rheumatology patients in routine care for anxiety, depression and FM using the MDHAQ or comparable questionnaire to enhance clinical decision-making and longitudinal care.

## Data Availability

Data are available on reasonable request to the corresponding author.
